# The Beaumont Meeting

**Published:** 1898-01

**Authors:** 


The Beaumont Meeting.—The South Texas Medical Society held its regular meeting at Beaumont on the 28th of December (ult.), as per announcement in our last issue. It was very largely attended, there being physicians there from every part of South and Middle Texas. The Journal is much gratified to note the action taken on the "doctor's tax." It was as follows:

"It is the sense of this meeting that the occupation tax including physicians passed by the last legislature is unjust and unconstitutional, in that it bears unequally upon all."

It is particularly gratifying to the writer to note that these physicians, who were on the ground in the midst of the trouble, and who had every opportunity of seeing the effects of quarantine, endorsed so unqualifiedly the present system of State quarantine. On motion of Dr. R. Rutherford, Ex-State Health Officer, the following resolution was unanimously adopted. It is to be hoped that our congressmen may be induced by this emphatic expression of the physicians of Texas, to let us alone in the matter of State regulation of quarantine:

"*Resolved*, That we this day, as a body of medical men, decline to indorse any change from the present status of quarantine affairs, and under no circumstances do we consent to transfer to the United States Marine Hospital Service the right to govern and the State control of such service. It is further ordered that the secretary mail to representatives in the legislature a copy of this resolution."

For the meeting of the State Medical Association at Houston, next April, Dr. R. T. Morris was appointed chairman of committee of arrangements. Dr. B. F. Calhoun, of Beaumont, was elected president; Drs. J. W. Scott, of Houston, and T. W. Shearer, of Wallisville, vice-presidents; Dr. Vard H. Hulen, of Galveston, secretary and treasurer.

